# Circulating immune complexes in dogs with *Leishmania infantum* infection in a non-endemic country

**DOI:** 10.1371/journal.pone.0345948

**Published:** 2026-04-16

**Authors:** Melanie Kaempfle, Katrin Hartmann, Yury Zablotski, Roswitha Dorsch, Nuria Parody, Jerónimo Carnés, Michèle Bergmann

**Affiliations:** 1 LMU Small Animal Clinic, Centre for Clinical Veterinary Medicine, LMU Munich, Munich, Germany; 2 R&D Unit Allergy and Immunology, LETI Pharma S.L.U., Madrid, Spain; Academic Medical Center: Amsterdam UMC Locatie AMC, NETHERLANDS, KINGDOM OF THE

## Abstract

Circulating immune complexes (CIC) cause different organ lesions in canine leishmaniosis. This study aimed to measure CIC in dogs infected with *Leishmania (L.) infantum* in a non-endemic country and to analyze associations with disease parameters. Measurement of *Leishmania*-specific CIC was performed by a polyethylene glycol ELISA (cut-off: 0.274 optical density (OD)) every three months during a one-year study period in 52 *L. infantum*-infected dogs. Each appointment included a physical examination, complete blood count, serum biochemistry including C-reactive protein, urinalysis, *L. infantum* PCR and antibody ELISA. Statistical analyses included Mann-Whitney U tests, a multivariable robust linear regression, Spearman correlation, univariable logistic regression, and a receiver operating characteristic (ROC) curve. CIC levels differed significantly between dogs with and without lymphadenopathy (*p* < 0.01; *β*=−0.38; 95% CI: −0.52, −0.24), uveitis (*p* < 0.01; *β=−*0.52; 95% CI: −0.89, −0.15), seborrhea/hypotrichosis (*p* = 0.04; *β*=−0.11; 95% CI: −0.21, −0.01), and skin nodules (*p* < 0.01; *β* = 0.22; 95% CI: 0.13, 0.31). Significant moderate to strong correlations with CIC levels were found for *L. infantum* antibodies (*p* < 0.01; *r*_s_ = 0.65; 95% CI: 0.57, 0.72), globulin (*p* < 0.01; *r*_s_ = 0.60; 95% CI: 0.51, 0.68), albumin-to-globulin ratio *(p* < 0.01; *r*_s_=−0.56; 95% CI: −0.65, −0.47) and total protein (*p* < 0.01; *r*_s_ = 0.46; 95% CI: 0.35, 0.56). CIC levels were significantly higher in dogs with positive lymph node PCR (*p* = 0.04) as well as in dogs with disease relapses (*p* < 0.01). The ROC curve analysis revealed one highly specific cut-off value at 1.668 OD for differentiating between dogs with and without disease relapse (98% specificity; 60% sensitivity). Measurement of *Leishmania*-specific CIC is useful for monitoring dogs with *L. infantum* infections and for indicating disease relapse.

## Introduction

*Leishmania (L.) infantum* infections in dogs can have asymptomatic courses or cause clinical signs with different organ lesions that are commonly associated with a poor prognosis [1]. Decisive for the course of infection is the dog’s immune system; a predominantly Th1-mediated cellular immune response is necessary to counteract the intracellular pathogens’ replication and prevent establishment of (systemic) disease. In contrast, a predominantly Th2-mediated humoral immune response favors the emergence of disease signs; polyclonal B cell activation leads to an excessive production of non-protective immunoglobulins, which in turn contribute to the formation of circulating immune complexes (CIC), consisting of (formerly free) *Leishmania* antigen, immunoglobulin (Ig) G and/or IgM and partially of complement components [2–6]. The deposition of CIC in capillary walls and complement activation can lead or contribute to certain disease complexes (e.g., glomerulonephritis, uveitis, arthritis, vasculitis) [7–12]. In general, the deposition of CIC and the resulting inflammatory response is considered to be one of the main pathological mechanisms leading to clinical signs in dogs with leishmaniosis, along with other inflammatory reactions (acute phase reaction, granulomatous/lymphoplasmacytic inflammation) and autoantibody production [4,13–15]. CIC might also enhance interleukin-10 expression by macrophages and thus, lower the capacity of intracellular parasite clearance, as shown in experimentally infected mice [16].

For the detection of CIC, a physical separation from the sample, e.g., with polyethylene glycol (PEG) or chemical binding of Fc regions or complement components contained in CIC are applied [17,18]. Since also different other etiologies can cause CIC formation, e.g., infections with *Dirofilaria (D.) immitis*, *Ehrlichia (E.) canis,* or autoimmune diseases, specific identification of the CIC is necessary [17,19–22].

A few, mainly preliminary studies were conducted on *Leishmania*-specific CIC in countries endemic for canine leishmaniosis [23–25]. Data of dogs living in non-endemic countries are missing. The advantage of studies in non-endemic countries, however, is the exclusion of re- or super-infection. Therefore, the aims of this prospective study were to measure CIC levels in naturally *L. infantum*-infected dogs in a non-endemic country, to further investigate their association with different disease parameters and to determine their use for monitoring dogs with *L. infantum* infection, especially to identify disease relapse.

## Materials and methods

### Study population

CIC measurement was part of a prospective clinical study authorized by the ethical committee of the Centre for Clinical Veterinary Medicine of the LMU Munich (Government of Upper Bavaria, reference number 244-06-12-2020 and 311-09-06-2022). It was performed between 2021 and 2023 and included 52 privately-owned dogs presented to the LMU Small Animal Clinic, Munich, Germany for regular routine rechecks as part of their ongoing management of *Leishmania* infections. Some of the samples were part of another study [26]. Inclusion criteria required proof of *Leishmania* infections by positive antibody (ELISA/IFAT) and/or PCR tests prior to enrollment. To avoid potential re-exposure to *Leishmania* parasites, owners were required not to take their dogs to any endemic country during the one-year study period. Furthermore, owners agreed to attend study appointments every three months. Dogs were only considered for enrollment if (maintenance) treatment with allopurinol was applied. The enrollment of dogs included an abdominal ultrasound examination and a qualitative point-of-care (POC) test (SNAP^®^ 4Dx Plus, IDEXX Laboratories Inc., Westbrook, ME, USA) to screen for comorbidities and coinfections with *E. canis, Borrelia burgdorferi, Anaplasma phagocytophilum/platys,* and *D. immitis*. Dogs were not enrolled in case of severe comorbidities, untreated *D. immitis* or *E. canis* infections, and/or systemic immunosuppressive treatment.

During the study, antileishmanial treatment was applied following dosing schedules recommended in current literature [27–30]. Allopurinol was commonly administered at 10 mg/kg, q12h, PO (with consideration of dose reduction in case of adverse urinary tract events) and owners were advised to feed low-purine diets to avoid development of xanthine urolithiasis [29,31]. In dogs that received leishmanicidal treatment, miltefosine (2 mg/kg, q24h, PO) or meglumine antimoniate (100 mg/kg, q24h, SC) were applied for 4 weeks. Additional symptomatic treatment was given to the dogs as needed.

### Study appointments

The dogs were monitored during a one-year study period that covered five study appointments at three-month intervals at the LMU Small Animal Clinic. Each appointment comprised a thorough physical examination, non-invasive blood pressure monitoring, blood sampling, conjunctival swab collection and/or fine-needle aspiration of lymph node, ultrasonographic examinations of the urinary tract and urine sampling (preferably by cystocentesis). Laboratory analyses included complete blood count (CBC) with leukocyte differential, serum biochemistry including symmetric dimethylarginine (SDMA) and C-reactive protein (CRP), urinalysis including urine protein-to-creatinine ratio (UPC), quantitative *Leishmania* PCR of conjunctival swabs and/or lymph node aspirates, an ELISA for *L. infantum* antibodies and a PEG-(IgG)-ELISA for *Leishmania*-specific CIC.

For CBC, an automated in-house device (Sysmex XT-2000iV; Sysmex Corporation, Kobe, Japan or ProCyte Dx, IDEXX Laboratories Inc., Westbrook, ME, USA) was used. Urine specific gravity was determined by an optical refractometer. Urine dipsticks were analyzed (IDEXX UA test strips read by the IDEXX UA Analyzer; IDEXX Laboratories Inc., Westbrook, ME, USA) and urine sediment analysis performed with an automated in-house analyzer (SediVue, IDEXX Laboratories Inc., Westbrook, ME, USA).

Serum biochemistry, urine protein analysis (UPC), *Leishmania* PCR and antibody ELISA were performed at an external laboratory (IDEXX GmbH, Kornwestheim, Germany). For shipment, which was performed on the day of sampling, clotted blood samples were centrifugated. Serum samples, aliquots of urine, conjunctival swabs, and/or swabs containing lymph node aspirate were placed in isolated containers together with cooling packs. Any surplus material, including serum samples used for CIC measurement, was stored at −80 °C until further analysis. Different disease parameters were assessed at each timepoint to investigate their associations with CIC levels (Table 1).

**Table 1:** Disease parameters assessed at each study appointment to investigate associations with circulating immune complexes.

Clinical findings	Complete blood count	Biochemistry	Urinalysis	Parasitological parameters
pale mucous membranes	non-regenerative anemia	hyperproteinemia	proteinuria^*^	*L. infantum* antibodies
lymphadenopathy	*hct < 35/37.3% (H1/2)*	*tp > 7.6 g/dl*	*UPC*^*b*^ > 0.5	*ELISA units>12*
arthritis	thrombocytopenia	hyperglobulinemia		*L. infantum* load (ln)
uveitis	*plt < 150/148 × 10⁹/L (H1/2)*	*glob>4.3 g/dl*		*parasites/10*^*6*^ *cells*
seborrhea/hypotrichosis	neutropenia	hypalbuminemia		
papules/nodules	*neut<3.00/2.95 × 10⁹/L (H1/2)*	*alb < 2.8 g/dl*		
skin ulcers	lymphopenia	low A/G ratio		
	*lymph<1.00/1.05 × 10⁹/L (H1/2)*	*A/G ≤ 0.8*		
	monocytosis	renal azotemia		
	*mono>0.5/1.12 × 10⁹/L (H1/2)*	*crea*^*a*^ ≥ 1.4 *mg/dl*		
		increased CRP		
		*CRP > 10.7 mg/l*		

alb, albumin; A/G ratio, albumin-to-globulin ratio; crea, creatinine; CRP, C-reactive protein; glob, globulins; hct, hematocrit; H1, automated hematological analyzer 1 (Sysmex XT-2000iV; Sysmex Corporation, Kobe, Japan); H2, automated hematological analyzer 2 (ProCyte Dx, IDEXX Laboratories Inc., Westbrook, ME, USA); *L. infantum*, *Leishmania infantum*; ln, lymph node; lymph, lymphocytes; mono, monocytes; neut, neutrophils; plt, platelets; tp, total protein; UPC, urine protein-to-creatinine ratio; ^a^, censored for correlation analysis if increased without renal azotemia; ^b^, censored for correlation analysis (UPC > 0.1) in case of active sediment (>5 leukocytes and/or epithelial cells per high power field, bacteria and/or spermatozoa) or macroscopic hematuria; *censored for Mann-Whitney U test in case of 0.5 < UPC < 2.0 and active sediment or macroscopic hematuria

### PEG-ELISA for CIC measurement

CIC measurement was performed with a previously validated modified precipitation ELISA method at the R&D Unit of LETI Pharma, Madrid, Spain [23,24] using surplus serum samples that were stored at −80 °C until analysis and shipped with dry ice. *Leishmania*-specific ELISA was performed and results were considered positive if optical density (OD) read at 492 nm exceeded 0.274, a cut-off value previously established (mean OD of negative samples +3 standard deviation) [24].

### Disease relapse

Disease relapse was defined as an onset or worsening of clinical and/or laboratory disease parameters occurring for the first time or occurring after previous either complete or partial remission following therapeutic interventions.

For evaluation of an association between disease relapse and CIC levels, each dog was considered only once; in dogs without disease relapse during the study period, CIC levels of the first appointment at which allopurinol was administered for at least three months without concomitant leishmanicidal treatment were evaluated. In dogs with repeated disease relapses, CIC levels from the time of the first relapse occurrence within the study period were evaluated. Associations with a CIC cut-off value (1.539 OD) previously proposed by Sarquis et al. (2024) [25] for the diagnosis of disease relapse were investigated.

### Statistical analysis

R statistical software (version 4.4.1.) was used for statistical analysis. All appointments were treated as independent events. Group comparisons of CIC levels between dogs with and without certain clinical signs were made using Mann-Whitney U tests (because of non-normal distribution, as assessed by Shapiro-Wilk normality test), which were followed by multivariable robust linear regression. All parameters that were either significantly associated (*p* < 0.05) or showed a trend toward significant association (0.05 < *p* < 0.1 [32]) with CIC levels in univariable analysis were considered for multivariable analysis. Multivariable analysis included manual backward selection.

Associations between CIC levels and various laboratory parameters were investigated using Mann-Whitney U tests due to non-normal-distribution of data (Shapiro-Wilk normality test) and Spearman’s rank correlation due to a violation of parametric assumptions. Significant correlations (*p* < 0.05) were considered moderate to strong if the correlation coefficient (*r*ₛ) exceeded 0.4 or was below −0.4 [33].

For investigations into associations between CIC and dogs with disease relapses, the Mann-Whitney U test was used due to a non-normal distribution of the data (Shapiro-Wilk normality test). Associations with a previously proposed cut-off value for the differentiation of dogs with and without disease relapse [25] were analyzed by univariable logistic regression. A receiver operating characteristic (ROC) curve and the area under the curve (AUC) were calculated for establishing a relapse cut-off value based on the present study population. Data were bootstrapped and the Youden index was maximized with a tolerance of 0.03.

## Results

### Dog population and study course

The study population consisted of 52 dogs aged between 11 months and 14 years. All dogs originated from or had travelled to areas endemic for canine leishmaniosis and had been diagnosed to be infected with *L. infantum* prior to inclusion. Thus, dogs were enrolled at different stages of *Leishmania* infections. Detailed baseline characteristics are provided in Table 2.

**Table 2:** Baseline characteristics of the study population.

Characteristic	Total (%)
**Sex**	
male	20 (38.5)
female	32 (61.5)
**Breed**	
purebred	16 (30.8)
mixed breed	36 (69.2)
**LeishVet classification**	
stage I	33 (63.5)
stage II	7 (13.5)
stage III	9 (17.3)
stage IV	3 (5.8)

Dogs were classified according to the LeishVet guidelines [34].

**Fig 1:**
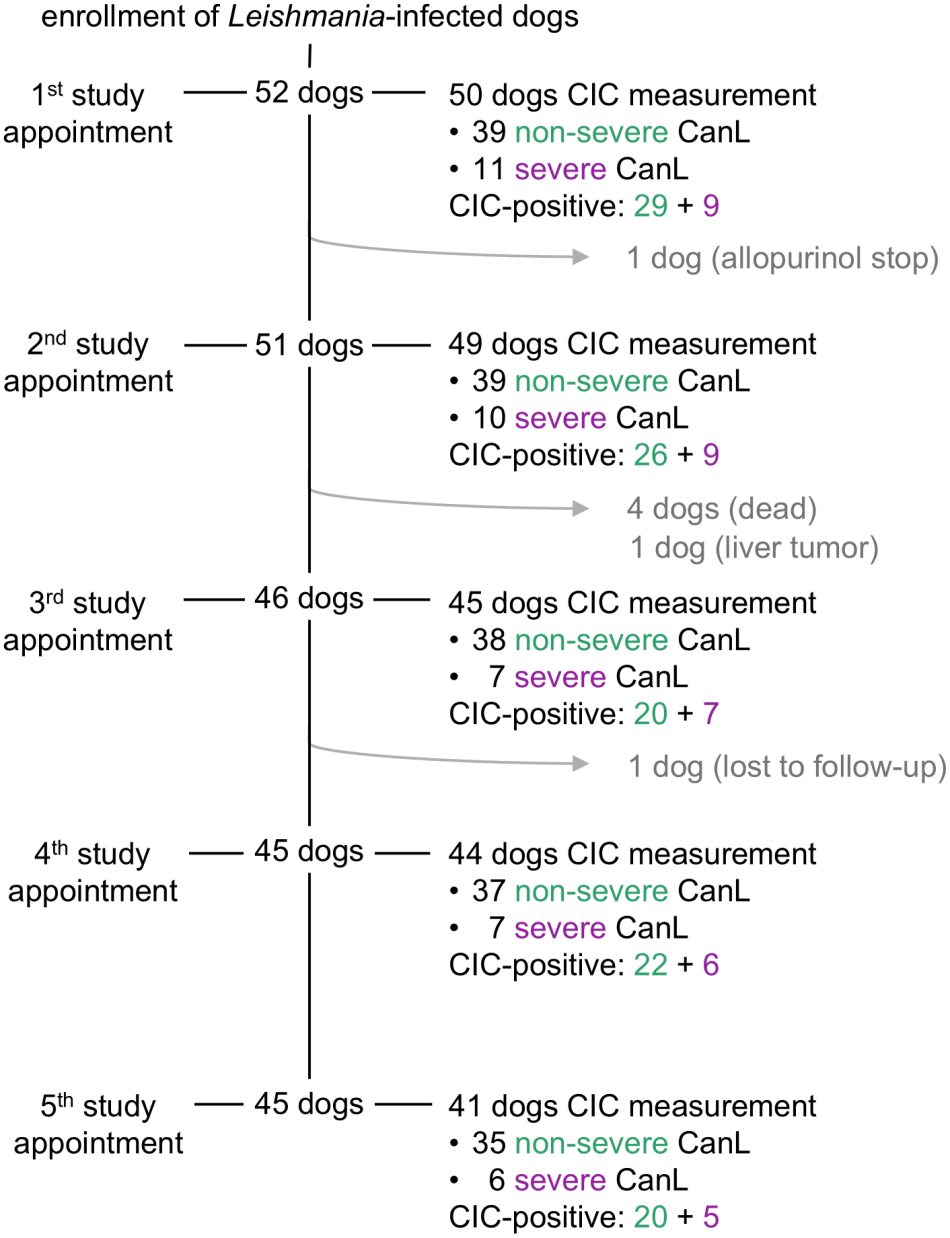
Overview of dogs enrolled and followed-up during the one-year study period. Dogs attended study appointments every three months during one year and were classified into non-severe cases (LeishVet stages I and II) and severe cases (LeishVet stages III and IV) [34]. One dog was excluded after the first study appointment due to termination of allopurinol treatment. After the second study appointment, one dog was euthanized due to critical worsening of non-regenerative anemia and azotemia during a recurrent disease relapse only a few months after the last leishmanicidal treatment. One dog that suffered from end stage chronic kidney disease was euthanized, two dogs died peracutely of unknown causes and one dog was excluded due to diagnosis of a liver tumor. Another dog was lost to follow-up after the third study appointment. CanL, canine leishmaniosis; CIC, circulating immune complexes; CIC-positive, polyethylene glycol ELISA results >0.274 optical density read at 492 nm.

In total, 45/52 dogs completed the one-year study period and attended all five scheduled study appointments, whereas the remaining seven dogs could not complete the study for various reasons (Fig 1).

### CIC levels and associations with different disease parameters

Measurement of CIC was performed on samples obtained at 229 appointments (Fig 1). Serum samples from ten appointments were not available for CIC measurement. Results of the PEG-ELISA ranged between 0.061 OD and 3.296 OD (median 0.382 OD). A total of 153/229 (66.8%) samples were positive (OD > 0.274).

CIC levels differed depending on the presence or absence of individual clinical signs. By univariable analysis (Mann-Whitney U test), significant differences in CIC levels were observed between dogs with and without lymphadenopathy (*p* < 0.01), uveitis (*p* < 0.01), seborrhea/hypotrichosis (*p* < 0.01), skin ulcers (*p* < 0.01) and pale mucous membranes (*p* < 0.01). Based on the preselection threshold (*p* < 0.1), skin papules/nodules (*p* = 0.06) were also included in the multivariable robust linear regression, whereas arthritis was excluded from further analyses (*p* = 0.45). The subsequent robust linear regression identified significantly higher CIC levels in dogs with lymphadenopathy (*p* < 0.01; *β=−*0.38; 95% CI: *−*0.52, *−*0.24), uveitis (*p* < 0.01; *β=−*0.52; 95% CI: −0.89, −0.15) and seborrhea/hypotrichosis (*p* = 0.04; *β=−*0.11; 95% CI: −0.21, −0.01) compared to dogs without the respective clinical signs. In contrast, dogs with skin papules/nodules had significantly lower CIC levels than those without (*p* < 0.01; β = 0.22; 95% CI: 0.13, 0.31) (Table 3).

**Table 3:** Clinical signs analyzed for associations with circulating immune complex levels by Mann-Whitney U test and multivariable robust linear regression.

Parameter		*n*	CIC level (OD)			UVA	MVA		
			Median	Range		*p-*value	*β*	95% CI	*p*-value
lymphadenopathy	yes	45	0.851	0.117	3.296	<0.01	–	–	<0.01
	no	184	0.333	0.061	2.843		−0.38	−0.52, −0.24	
uveitis	yes	7	1.134	0.465	1.864	<0.01	–	–	<0.01
	no	222	0.366	0.061	3.296		−0.52	−0.89, −0.15	
seborrhea/hypotrichosis	yes	88	0.558	0.098	3.296	<0.01	–	–	0.04
	no	141	0.333	0.061	2.096		−0.11	−0.21, −0.01	
papules/nodules	yes	13	0.209	0.066	1.370	0.06	–	–	<0.01
	no	216	0.390	0.061	3.296		0.22	0.13, 0.31	
skin ulcers	yes	14	1.080	0.098	2.605	<0.01	removed by backward selection		
	no	215	0.363	0.061	3.296				
pale mucous membranes	yes	5	1.645	0.701	2.038	<0.01	removed by backward selection		
	no	224	0.367	0.061	3.296				
arthritis	yes	2	0.548	0.531	0.564	0.45	n.a.		
	no	227	0.373	0.061	3.296				

Regression coefficients (*β*) indicate standardized changes in circulating immune complex levels associated with the respective disease parameters in the multivariable robust linear regression.

CIC, circulating immune complexes; CI, confidence interval; MVA, multivariable analysis; *n*, number of dogs per category; n.a., not applicable; OD, optical density read at 492 nm; UVA, univariable analysis

**Fig 2:**
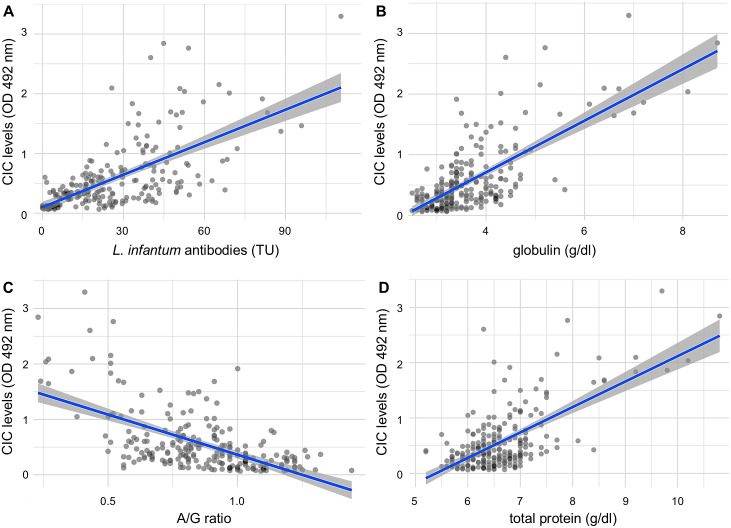
Laboratory parameters significantly correlated (p < 0.05) with circulating immune complex levels measured as optical density (OD) at 492 nm in a modified polyethylene glycol ELISA, with Spearman correlation coefficient (rs) greater than 0.4 or less than −0.4. (A) *Leishmania infantum* antibody level measured by ELISA: *r_s_* = 0.65; (B) serum globulin concentration: *r_s_*   = 0.60; (C) albumin-to-globulin (A/G) ratio: *r_s_*  =−0.56; (D) total protein: *r_s_*   = 0.46; CIC, circulating immune complexes; OD, optical density; TU, test units.

For laboratory parameters, associations with CIC levels were investigated by correlation analysis (Table 4). CIC levels showed significant but rather low correlation (*r*_s_ between −0.4 and 0.4) with levels of creatinine (*r*_s_ = 0.19; *p* < 0.01), UPC (*r*_s_ = 0.21; *p* < 0.01), albumin (*r*_s_=−0.35; *p* < 0.01), hematocrit (*r*_s_=−0.36; *p* < 0.01) and CRP (*r*_s_ = 0.36; *p* < 0.01) and moderate to high correlations with total protein (*r*_s_ = 0.46; *p* < 0.01), A/G ratios (*r*_s_=−0.56; *p* < 0.01), globulin (*r*_s_ = 0.60; *p* < 0.01), and *L. infantum* antibody levels (*r*_s_ = 0.65; *p* < 0.01) (Fig 2). Since the biochemical parameters that were found to have moderate to strong correlations with CIC levels were highly collinear (variance inflation factor > 10), a multivariable analysis was not performed.

**Table 4:** Laboratory parameters analyzed for correlations with circulating immune complex levels using Spearman’s rank correlation.

Parameter	*n*	Median	Range		Correlation with CIC		
					*r*_*s*_	95% CI	*p*-value
**hematocrit (%)**	229	46.7	21.2	60.6	**−0.36**	−0.47, −0.24	**<0.01**
thrombocytes (10⁹/L)	227	241	82	791	0.01	−0.12, 0.15	0.86
monocytes (10⁹/L)	229	0.31	0.06	1.26	0.09	−0.04, 0.22	0.18
neutrophils (10⁹/L)	229	4.22	2.08	11.11	0.04	−0.10, 0.17	0.56
lymphocytes (10⁹/L)	229	1.95	0.69	4.16	0.02	−0.11, 0.16	0.74
**creatinine (mg/dl)**	225	0.9	0.3	4.7	**0.19**	0.06, 0.32	**<0.01**
**total protein (g/dl)**	229	6.0	5.2	10.8	**0.46**	0.35, 0.56	**<0.01**
**albumin (g/dl)**	229	3.0	1.6	3.7	**−0.35**	−0.46, −0.22	**<0.01**
**globulin (g/dl)**	229	3.4	2.5	8.7	**0.60**	0.51, 0.68	**<0.01**
**A/G ratio**	229	0.85	0.23	1.44	**−0.56**	−0.65, −0.47	**<0.01**
**CRP (mg/l)**	229	3.8	0.3	67.1	**0.36**	0.24, 0.47	**<0.01**
**UPC**	210	0.1	0.1	17.6	**0.21**	0.07, 0.34	**<0.01**
***L. infantum* antibodies (TU)**	229	21.8	0.1	110.5	**0.65**	0.57, 0.72	**<0.01**

The correlation coefficient (*r*_s_) indicates the strength of association (moderate to strong correlation if *r*_s_ above 0.4 or below −0.4). Bold type indicates parameters significantly associated with circulating immune complex levels in correlation analysis (*p* < 0.05). A/G ratio, albumin-to-globulin ratio; CIC, circulating immune complexes; CRP, canine C-reactive protein; *L. infantum*, *Leishmania infantum;* TU, test unit; UPC, urine protein-to-creatinine ratio

### CIC levels and *Leishmania* load

**Fig 3:**
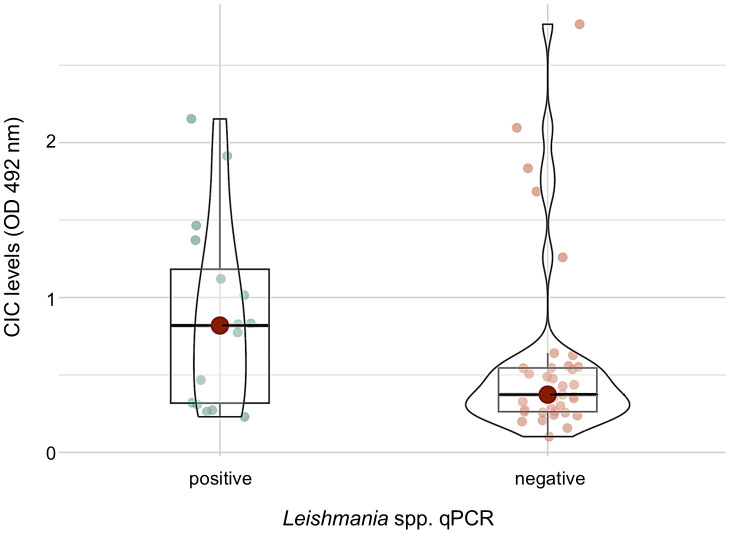
Comparison of circulating immune complex levels of dogs with positive (*n* = 16) and negative (*n* = 37) *Leishmania* spp. qPCR of lymph node aspirates. The boxplots depict the distribution of CIC levels given in optical density (OD) determined at 492 nm in a modified polyethylene glycol ELISA. The central line within each box represents the median; box limits indicate the interquartile range (IQR), and whiskers extend to 1.5 × IQE. Dots beyond this range represent outliers. Dogs with positive qPCR had significantly higher CIC levels (median 0.82 OD) than dogs without (median 0.37 OD) (*p =* 0.04; Mann-Whitney U test).

A total of 53 lymph node aspirate samples were available for qPCR analysis; in positive samples (*n* = 16), *Leishmania* load ranged between 1–70,700 parasites/1 million cells. There was a significant difference (*p* = 0.04) in CIC levels of dogs with positive (*n* = 16; median: 0.82 OD) and dogs with negative (*n* = 37; median: 0.37 OD) qPCR results (Fig 3), but no significant correlation (*p* = 0.99) was found between the parasite load of positive samples and CIC levels.

### CIC levels and disease relapse

**Fig 4:**
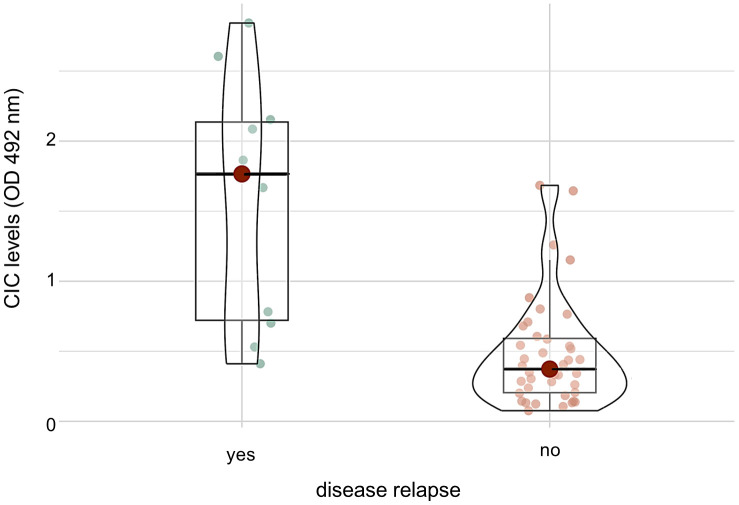
Comparison of circulating immune complex levels of dogs with (n = 10) and dogs without (n = 40) disease relapse. The boxplots depict the distribution of circulating immune complex (CIC) levels given in optical density (OD) determined at 492 nm in a modified polyethylene glycol ELISA. The central line within each box represents the median; box limits indicate the interquartile range (IQR), and whiskers extend to 1.5 × IQE. Dots beyond this range represent outliers. Dogs with disease relapse had significantly higher CIC levels (median 1.766 OD) than dogs without disease relapse (median 0.373 OD) (p < 0.01; Mann-Whitney U test).

To determine an association between CIC levels and disease relapse, samples from 50 dogs (10/50 with and 40/50 without relapse) were available. CIC levels of dogs with disease relapse ranged between 0.411 to 2.843 (Table 5), those of dogs without relapse between 0.076 and 1.684 OD. The median CIC level was significantly higher in dogs with disease relapse (1.766 OD) than in dogs without (0.373 OD) (Fig 4).

**Table 5:** Circulating immune complex levels and main clinical and laboratory findings of dogs with disease relapse.

Dog	CIC	Main findings			
No.	OD	Clinical signs	CBC	Biochemistry	Others
1	2.843	mild seborrhea	anemia, thrombocytopenia, lymphopenia	hypalbuminemia, hyperglobulinemia, low A/G ratio, hyperproteinemia, increased CRP	proteinuria, *Leishmania* antibodies and PCR (cs) positive
2	2.605	skin ulcer, mild seborrhea	lymphopenia	hypalbuminemia, hyperglobulinemia, low A/G ratio, increased CRP	proteinuria, *Leishmania* antibodies positive
3	2.153	skin ulcer, mild seborrhea/hypotrichosis, lymphadenopathy	thrombocytopenia, neutropenia, lymphopenia	hypalbuminemia, hyperglobulinemia, low A/G ratio, hyperproteinemia, increased CRP	*Leishmania* antibodies and PCR (ln) positive
4	2.086	skin ulcers, seborrhea/hypotrichosis	monocytosis	hypalbuminemia, hyperglobulinemia, low A/G ratio, hyperproteinemia, increased CRP	proteinuria, *Leishmania* antibodies positive
5	1.864	uveitis, skin ulcers, seborrhea, lymphadenopathy	anemia	hypalbuminemia, hyperglobulinemia, low A/G ratio, hyperproteinemia, increased CRP	*Leishmania* antibodies positive
6	1.668	--	anemia, monocytosis	hyperglobulinemia, low A/G ratio, hyperproteinemia	*Leishmania* antibodies positive
7	0.782	lymphadenopathy, edema, skin ulcer, seborrhea/hypotrichosis	--	hypalbuminemia, hyperglobulinemia, low A/G ratio	proteinuria, *Leishmania* antibodies positive
8	0.701	lymphadenopathy	anemia, neutropenia	hypalbuminemia, low A/G ratio	proteinuria, *Leishmania* antibodies and PCR (cs) positive
9	0.531	arthritis, lymphadenopathy, skin papules, mild seborrhea	neutropenia, lymphopenia	--	*Leishmania* antibodies positive
10	0.411	--	thrombocytopenia, neutropenia	azotemia, hypalbuminemia, low a/g ratio	proteinuria, *Leishmania* antibodies positive

A/G ratio, albumin-to-gobulin ratio; CBC, complete blood count; CIC, circulating immune complexes; CRP, C-reactive protein; cs, conjunctival swab, ln, lymph node aspirate; No., number; OD, optical density read at 492 nm.

Dogs experiencing disease relapse in the present study were shown to have significantly higher odds (OR 20.1; *p* < 0.01) to exceed the CIC cut-off value (6/10 dogs) previously proposed by Sarquis et al. (2024) [25] for the diagnosis of disease relapse (1.539 OD) than dogs without disease relapse (2/40 dogs).

**Fig 5:**
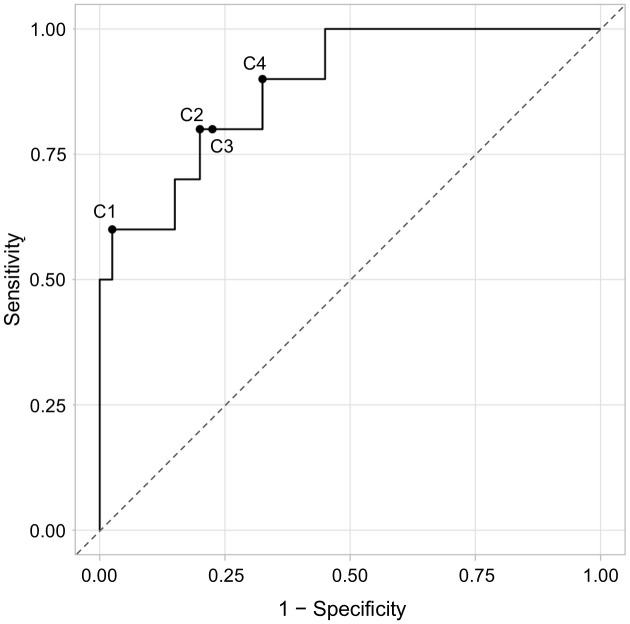
Receiver operating characteristic (ROC) curve illustrating the diagnostic performance of circulating immune complexes in distinguishing dogs with disease relapse from dogs without disease relapse. The area under the curve (AUC) of 0.885 indicates good discriminatory ability. Sensitivity and specificity were calculated across a range of cut-off values. The dashed line represents the line of no discrimination (AUC = 0.5). The dots represent the four optimal cut-off values (C1-4) according to the maximum Youden-Index (J): 1.668 OD, J = 0.575 (C1); 0.701 OD, J = 0.600 (C2); 0.681 OD, J = 0.575 (C3), 0.531 OD, J = 0.575 (C4).

The ROC curve analysis, which maximized Youden’s index via bootstrapping, was performed to determine a cut-off value for distinguishing between dogs with and without disease relapse based on the present study population. It revealed an AUC of 0.885 and four possible cut-off values (C1-4), at 1.668 OD (97.5% specificity; 60% sensitivity), 0.701 OD (80.0% specificity; 80% sensitivity), 0.681 (77.5% specificity; 80% sensitivity) and 0.531 OD (67.5% specificity; 90% sensitivity) (Fig 5). Bootstrap analysis revealed a broad interquartile range (0.53–1.65 OD), with a median cut-off at 0.70 OD and two frequently selected cut-off regions across bootstrap iterations, clustering around the lowest three and the highest identified cut-off values (*n* = 1000 replicates).

## Discussion

*Leishmania*-specific CIC are assumed to circulate in the bloodstream and, in case of accumulation, get deposited in capillary walls of different tissues, resulting in local inflammatory reactions [2,17]. Thus, dogs presenting with signs attributable to the deposition of immune complexes, such as glomerulonephritis, uveitis, arthritis and vasculitis are expected to have high CIC levels in blood. It was also hypothesized that due to tissue deposition, CIC levels in blood might decline [35–37]. This might, however, be difficult to prove since the chronic antigen stimulation in canine leishmaniosis and an associated excessive humoral immune response might maintain steady CIC blood levels, at least in untreated dogs or in case of reinfections. In endemic countries, levels of *Leishmania*-specific CIC were shown to correlate with disease severity of naturally infected dogs in a preliminary study [23,24]. In a prospective clinical trial, CIC levels differed significantly between dogs with and without concurrent disease relapse, for which a CIC cut-off value was calculated (1.539 OD) and proposed for evaluation in further studies [25]. The present study measured CIC levels in dogs in a non-endemic country and also observed significant differences in CIC levels of dogs with and without disease relapse (median 1.766 OD and 0.373 OD, respectively). Detection of relapse can be challenging in dogs presenting with a rather gradual (than sudden) deterioration along with inconclusive clinical and/or laboratory findings [38–41]. Likewise, challenges in early detection of relapse diagnosis emerge if dogs present with (new) signs that cannot be clearly attributed to leishmaniosis or potential comorbidities [42–45].

The relapse cut-off value recently proposed by Sarquis et al. (2024) [25] was evaluated in the present study population; dogs with disease relapse had approximately 20-fold higher odds for CIC levels above this threshold than dogs without disease relapse. The proposed cut-off, however, was associated with a rather low specificity (71%) in the study by Sarquis et al. (2024) [25]. Since detection of relapse is commonly followed by leishmanicidal treatment, high specificity is crucial to avoid any unnecessary treatment, especially in the light of related risks for adverse effects and drug resistance [46–48]. The ROC curve analysis performed with the data of the present study population revealed four different cut-off values for identification of disease relapse, varying considerably in specificity (68–98%) and sensitivity (60–90%). The cut-off with the highest specificity in the present study was 1.668 OD (98% specificity; 60% sensitivity).

Thus, although only a little higher (+0.129 OD) than the cut-off previously proposed by Sarquis et al. (2024) [25], it was associated with a considerably higher specificity. The difference in specificity might result from deviations in the study design. In the present study, each dog was considered only once for cut-off analysis. Further, blood samples from dogs right at the end of their leishmanicidal treatment were not considered; a decrease of CIC levels at this timepoint was not observed in naturally infected dogs in Spain [25] and dogs from the present study, which resembles findings on antibody dynamics after treatment [49–51]. Thus, CIC results always need to be interpreted in view of the dog’s treatment history. Nevertheless, further studies evaluating time-dependent CIC variations following treatment are needed. Besides leishmanicidal treatment, the impact of immunosuppressive drugs, which can be indicated in dogs with CIC-related manifestations, such as proteinuria, uveitis, arthritis, or vasculitis would be of particular interest [25,29,52].

In accordance with the rather low sensitivity (60%) of the cut-off at 1.668 OD, relapses remained unidentified in 4/10 dogs in the present study. This is to some extent surprising, since all these dogs presented with signs that are commonly related to immune complex deposition (i.e., glomerulonephritis and arthritis). However, indeed, although dogs with glomerulonephritis and proteinuria might have higher CIC levels than dogs without [25], only low correlation between the degree of proteinuria and CIC levels was found in the present study. It is possible that previous CIC deposition led to irreversible glomerular damage in the past [7], probably reflected in persistent proteinuria but not in high CIC levels anymore. Furthermore, immune complex deposits consisting of IgM and IgA, which cannot be detected in blood by the modified IgG-ELISA used in the present study, might have contributed to glomerulonephritis [37,53–56]. Glomerulonephritis in dogs with leishmaniosis was also shown to be associated with a glomerular infiltration of T cells and involvement of adhesion molecules, but the extent of their contribution to proteinuria needs to be further evaluated [29,40,57]. It is furthermore possible that symptomatic antiproteinuric treatment, which is indicated in dogs with glomerulonephritis [56,58], limits conclusions on CIC results. Further investigations of CIC and UPC, e.g., in patients undergoing immunoadsorption, a hemodialysis-related extracorporeal technique for the removal of (*Leishmania*-specific) CIC from blood circulation [59], are needed to obtain insights into the dynamic association between CIC in blood and the degree of proteinuria. Furthermore, studies on CIC levels in dogs with untreated proteinuria and/or concomitant histopathological investigations of kidney biopsies would be valuable.

Interestingly, in the present study, dogs with uveitis had significantly higher CIC levels than dogs without uveitis. So far, there is no conclusive evidence about the origin of immune complexes deposited in the uvea; a local formation following local IgG production and ocular penetration of *Leishmania* parasites, as well as a derivation from blood circulation are discussed [11,40,60,61]. Since uveitis is not pathognomonic for canine leishmaniosis and observed also in different other infectious and non-infectious diseases [62,63], measurement of CIC levels might be helpful to determine its underlying etiology. In case of canine leishmaniosis, it has to be considered that uveitis can also emerge due to inflammatory response to the parasites or as a complication after anti-*Leishmania* treatment, probably due to a local allergic reaction to the destroyed parasites [10,64–66]; the contribution of CIC remains unclear. Thus, an evaluation of immune complex measurement in aqueous humour and comparison to levels in blood of dogs with uveitis would be of great value.

In the present study, significantly higher CIC levels were also observed in dogs with lymphadenopathy, a frequent problem in dogs with leishmaniosis [67]. Lymphadenopathy is attributable to lymphoid hyperplasia, resulting from B cell and plasma cell proliferation (usually polyclonal), alone or along with granulomatous inflammation resulting from histiocyte proliferation (i.e., dendritic cells and macrophages) [68–70]. Proliferation of B cells and a concomitant exuberant humoral immune response are commonly related to an enhanced CIC formation, which might explain the present study’s findings [13].

Significantly higher CIC levels were also found in dogs presenting with seborrhea and/or hypotrichosis in the present study. However, so far, there is no consensus about the (immune) pathogenesis of these rather unspecific clinical findings in dogs with canine leishmaniosis; a direct inflammatory response to the parasites themselves has been discussed [71]. However, at least in dogs with exfoliative dermatitis, parasite burden and histological findings of affected skin were comparable to those found in apparently healthy skin [72]. In addition, the skin of dogs with exfoliative dermatitis was found to be extensively infiltrated by B-cells and plasma cells, concluding that antibodies are involved in pathogenesis [73].

Significantly lower CIC levels were found in dogs with skin papules/nodules compared to those without in the present study. In fact, these signs emerge especially in dogs with a predominantly cellular immune response to parasitic infiltration, which are thought to maintain low levels of *Leishmania* antibodies and, obviously, also CIC blood levels [74].

In the present study, dogs with positive lymph node PCR results were found to have significantly higher CIC levels than dogs without, although no correlation was found between the parasite load and CIC levels. The latter finding was rather unexpected, since it is commonly assumed that CIC formation results from an excess of antigen over antibodies [29], and thus, a linear association between parasite load and CIC levels would be expected. Furthermore, dogs with an excessive humoral immune response, which might enhance CIC formation, were shown to be unable to control parasite replication, resulting in high parasitic burden [75]. Whether the discrepant finding of the correlation analysis resulted from leishmanicidal treatment, which might lead to a more rapid decrease in parasite load than in CIC levels, remains unclear [25,76,77].

With regard to laboratory parameters determined in blood samples of the dogs in the present study, high correlation between CIC levels and *L. infantum* antibody levels measured by an IgG-ELISA were found. This is in line with the findings of another study performed in an endemic country, in which *L. infantum* antibodies were measured by an IFAT [25]. This is reasonable, since the presence of antibodies is essential for the formation of CIC. Interestingly, 25/153 CIC-positive samples in the present study were negative or borderline in the concomitantly performed *L. infantum* IgG ELISA, likely because antibodies were complex-bound and thus not detectable [3,78,79]. To overcome this problem and increase sensitivity in the diagnosis of leishmaniosis, CIC measurement in addition to the commonly recommended antibody and PCR testing could be valuable [80].

A high correlation was also observed between CIC levels and serum globulin concentrations. This was, to some extent expected, since globulin concentrations largely depend on IgG concentrations and only to a lesser extent on other immunoglobulins (IgM, IgA, nonspecific Ig). Furthermore, it is hypothesized that CIC stimulate immunoglobulin production due to a lack of (complex-bound) antigen elimination and might thus contribute to hyperglobulinemia themselves [81]. Serum globulin concentrations (in turn) influence total serum protein concentrations [67], for which high correlations with CIC levels were also proven in the present study. Although commonly observed in dogs with *Leishmania* infections, hyperglobulinemia and hyperproteinemia are not pathognomonic and can also be observed in other chronic infectious or neoplastic diseases that are accompanied by enhanced antibody production [82,83]. Thus, hyperglobulinemia and hyperproteinemia in the absence of high *Leishmania*-specific CIC levels might be rather indicative for another underlying disease.

High correlations with CIC levels were also observed for the A/G ratio, a parameter indexing dysproteinemia, due to hyperglobulinemia and concomitant hypalbuminemia, resulting from negative acute phase reactions and/or proteinuria [41,67,84–86]. In contrast to biochemical parameters, none of the hematological parameters evaluated in the present study showed relevant correlations with CIC levels.

A limitation of the present study is that, due to its occurrence in only one dog, associations between CIC and arthritis could not been evaluated properly. However, since the deposition of CIC is one of the assumed underlying pathological mechanisms of *Leishmania*-related arthritis [9,87], insights into CIC levels in such dogs would be valuable.

## Conclusions

The findings of the present study demonstrate significant associations between CIC and different disease parameters in dogs with *L. infantum* infections living in a non-endemic area and further indicate the usefulness of CIC measurement especially for the identification of disease relapses. Thus, CIC measurement could help to diagnose and monitor dogs with *L. infantum* infections.
